# Independent risk factors for axillary lymph node metastasis in breast cancer patients with one or two positive sentinel lymph nodes

**DOI:** 10.1186/s12905-020-01004-7

**Published:** 2020-07-09

**Authors:** Wei Zhang, Jing Xu, Ke Wang, Xiao-Jiang Tang, Hua Liang, Jian-Jun He

**Affiliations:** 1 Department of Breast Surgery, The First Affiliated Hospital of Xi’an Jiaotong University, 277 Yanta West Rd., Xi’an, 710061 Shaanxi China; 2 Department of Geriatric Medicine, The First Affiliated Hospital of Xi’an Jiaotong University, Xi’an, 710061 Shaanxi China; 3 Department of Pathology, The First Affiliated Hospital of Xi’an Jiaotong University, Xi’an, 710061 Shaanxi China

**Keywords:** Breast cancer, Sentinel lymph node, Sentinel lymph node biopsy, Axillary lymph node, Axillary lymph node metastasis

## Abstract

**Background:**

The benefit of axillary lymph node dissection (ALND) in breast cancer patients with one or two positive sentinel lymph nodes (SLNs) remains inconclusive. The purpose of this study was to identify risk factors independently associated with axillary lymph node (ALN) metastasis.

**Methods:**

We retrospectively analyzed data from 389 Chinese breast cancer patients with one or two positive SLNs who underwent ALND. Univariate and multivariate logistic regression analyses were performed to identify ALN metastasis-associated risk factors.

**Results:**

Among the 389 patients, 174 (44.7%) had ALN metastasis, while 215 (55.3%) showed no evidence of ALN metastasis. Univariate analysis revealed significant differences in age (< 60 or ≥ 60 years), human epidermal growth factor receptor-2 (Her-2) status, and the ratio of positive to total SLNs between the ALN metastasis and non-metastasis groups (*P* < 0.05). The multivariate analysis indicated that age, the ratio of positive to total SLNs, and occupations were significantly different between the two groups. Lastly, younger age (< 60 years), a higher ratio of positive to total SLNs, and manual labor jobs were independently associated with ALN metastasis (*P* < 0.05).

**Conclusions:**

The risk of ALN metastasis in breast cancer patients with one or two positive SLNs can be further increased by younger age, manual labor jobs, and a high ratio of positive to total SLNs. Our findings may also aid in identifying which patients with one or two positive SLNs may not require ALND.

## Background

Breast cancer is the most common invasive malignancy detected in women around the globe, accounting for 22.9% of all female cancers [1–3]. In accordance to the World Health Organization (WHO), it has been estimated that 627,000 women died in 2018 from breast cancer worldwide, which is approximately 15% of cancer-related deaths in women [4]. Similar to other cancers, breast cancer cells can develop rapidly and become metastatic if left untreated or managed improperly in some cases, allowing cancer cells to spread to distant organs through nearby sentinel lymph nodes (SLNs), axillary lymph nodes (ALNs), or through other routes. The SLN is the node through which breast cancer cells spread from the original tumor site to the ALNs and other distant organs [5].

Sentinel lymph node biopsy (SLNB) was a landmark procedure that led to a fundamental shift in the management of the ALN metastases. In fact, SLNB has partly replaced the more traditional previous approach of axillary lymph node dissection (ALND) for the treatment of early-stage invasive breast cancer in some cases [6–14]. SLNB has also empowered physicians to assess disease stage, predict the prognosis of patients, and develop optimal treatment strategies for improving the quality of life and positive outcomes of patients [6, 10]. When the SLNB yields a positive finding, standard practices recommend excision of the ALNs during the SLNB procedure or scheduled follow-up procedures [11]. The SLNB may also help some breast cancer patients avoid unnecessary and highly invasive lymph node surgeries, such as ALND, which is a procedure associated with serious complications [11–14]. In contrast to ALND, SLNB has a lower incidence of procedure-related complications, including shoulder stiffness, pain, and lymphedema. Furthermore, removal of nearby lymph nodes may be unnecessary when the SLNB result is negative [11–14]. As all lymph node surgeries have some degree of risk, adverse effects of the procedure may be minimized if fewer lymph nodes are removed.

It has been proposed that a surgical procedure on extensive lymph nodes, such as ALND, may be avoided in breast cancer patients with negative SLNB findings [11–14]. Indeed, negative SLNB findings indicate that the breast cancer cells have not developed the ability to invade nearby lymph nodes in the body, while a positive SLNB result indicates that the cancer cells are present in the SLNs and may spread into nearby regional lymph nodes, and possibly distant organs of the body [5]. In addition, the value of ALND in breast cancer patients with one or two positive SLNs has been debated as omitting the procedure does not affect clinical outcomes [13]. In breast cancer patients with one SLN, the benefit of ALND has not been established. In addition, the incidence of ALN metastasis in breast cancer patients with one or two positive SLNs has not been largely studied in Chinese breast cancer patients. Therefore, it is important to identify risk factors associated with ALN status in breast cancer patients, particularly those with one or two positive SLNs who may or may not need immediate surgical intervention. Several previous studies indicated that a number of risk factors, such as age at the time of diagnosis, body mass index (BMI), tumor size, primary tumor quadrant, molecular subtype, estrogen receptor (ER) status, progesterone receptor (PR) status, human epidermal growth factor receptor-2 (Her-2) status, and other clinical parameters were associated with ALN metastasis in breast cancer [15–19]. However, the effects of these factors on the incidence of ALN metastasis were inconsistent due to ethnic/racial differences [19–23]. For this reason, researchers need a better understanding of the risk factors associated with ALN status, particularly in Chinese breast cancer patients with one or two positive SLNs.

In the present study, we retrospectively reviewed and analyzed data from Chinese breast cancer patients with one or two positive SLNs who had undertaken ALND with and without ALN metastasis. Univariate and multivariate logistic regression analyses were performed to determine the incidence of ALN metastasis in Chinese breast cancer patients with one or two positive SLNs and to identify risk factors independently associated with ALN metastasis. Our findings may allow predicting which patients are at high risk for ALN metastasis and may also lay the foundation for the development of an improved algorithm for predicting ALN status in breast cancer patients in the future.

## Methods

### Human subjects and study design

In this retrospective study, the demographic, social, laboratory tests, and clinical data of 389 Chinese breast cancer patients with one or two positive SLNs spanning the period between January 2010 to January 2018 at the First Affiliated Hospital of Xi’an Jiaotong University (Xi’an, Shaanxi, China) were reviewed and analyzed. The subjects who met the following inclusion criteria were consecutively enrolled in this study: (1) Primary cT1-2 N0 breast cancer was diagnosed and confirmed by pathological findings of a needle core biopsy; (2) presence of one or two positive SLNs; (3) ALND was performed. However, patients who had certain clinical conditions, such as bilateral breast cancer or metastasis to chest wall or other distant organs, were excluded from the study. From the 389 Chinese breast cancer subjects, 174 were diagnosed and confirmed to have ALN metastasis, while 215 showed no evidence of ALN metastasis. The study subjects were assigned to the two groups based on metastasis status with their demographic, social, laboratory tests, and clinical data. All patients in this study received post-surgery systemic chemotherapy in accordance with the local clinical practice guidelines.

The present study was approved by the Research Ethic Committee at the First Affiliated Hospital of Xi’an Jiaotong University (Xi’an, Shaanxi, China). Since this was a retrospective analysis of anonymous data, singed informed consent was waived by the committee. In addition, the study was carried out in compliance with the Declaration of Helsinki.

### Identification methods of SLNs

During pathological evaluation of the SLN metastasis, the SLNs were detected and visualized with an injection of methylthioninium chloride (Jiangsu Jumpcan Pharmaceutical Co., Ltd., Shanghai, China), radioactive substances such as nano-carbon suspension (Chongqing Lummy Pharmaceutical Co., Ltd., Chongqing, China), or indocyanine green (Liaoning Tianyi Biopharmaceutical Limited by Share Ltd., Shenyang, China). The injected materials were directly visualized (blue dyes) or assessed by radioactive imaging. SLNs were examined the blue/black-stained lymph vessels under the direction of a dye tracer. According to the manufacturer’s protocol, an SLN was defined as any blue/black-stained lymph node, any lymph node with a blue/black-stained lymphatic channel which led directly to it, any lymph node with a radioactive count of ≥10% of the most radioactive lymph node, or any pathologically palpable lymph node. Fast-frozen pathology was conducted on each SLN to check for the presence of breast cancer cells. Two expert pathologists reviewed the pathological findings. ALND was performed if the fast-frozen pathology revealed SLN metastasis with one or two positive lymph nodes.

### Histological examination and immunohistochemistry

All nodes were histologically examined with hematoxylin and eosin (H&E) staining. The status of Her-2, ER, and PR were assessed by immunohistochemistry (IHC) in combination with fluorescence in situ hybridization (FISH). The Her-2 positivity was defined as a score of 3+ alone or ≤ 2+ when combined with fluorescence in situ hybridization (FISH) findings [24–26].

### Univariate logistic regression analysis

The demographic, social, and clinicopathologic parameters, as well as laboratory tests findings, were used for the univariate logistic regression analysis to identify ALN metastasis-associated risk factors in Chinese breast cancer patients with one or two positive SLNs. For the univariate logistic regression analysis, the following demographic and social factors were included: age (< 60 or ≥ 60) and occupations, in which the study patients were categorized by occupational types into three groups: manual labor jobs (blue-color jobs with tasks requiring muscle power), mental labor jobs (white-color jobs such as teachers, librarians, and administrative jobs), and the other/rest (e.g. housekeeper, freelancer). In addition, the clinical, pathological, and laboratory tests parameters, which included menarche, menopausal status (pre-menopausal, post-menopausal as determined at the time of the initial diagnosis), delay in diagnosis (0–90, 91–180, 181–365, or ≥ 366 days), tumor primary site (right or left), tumor location (upper outer, lower outer, upper inner, or lower inner), tumor size (≤2 cm or >  2 cm), pathological type [invasive ductal carcinoma (IDC) or other], ER status (negative or positive), PR status (negative or positive), Her-2 status (negative or positive), triple negative status (yes or no), Ki-67 status (high or low based upon the cut-off value of 30%), tumor grade (1, 2, or 3), postoperative ALNs (≤10, 11–19, or ≥ 20) upon postoperative pathological examination, and the ratio of positive to total SLNs (≤0.05 or > 0.5) were integrated into the univariate logistic regression analysis to determine which of those were associated with the status of ALN metastasis in breast cancer patients with one or two positive SLNs.

### Multivariate logistic regression analysis

The demographic, social, and clinicopathologic characteristics were also subjected to a multivariate logistic regression analysis to determine independent risk factors for prediction of ALN metastasis in breast cancer patients with one or two positive SLNs. In addition, odds ratios (OR) with 95% confidence interval (CI) were calculated and presented.

### Statistical analysis

In this study, all variables except for age were categorical variables. Age was converted to a categorical variable for analysis. Categorical data were presented as frequencies and percentages. The Pearson’s chi-squared test (*χ*^2^) was used to assess differences in axillary lymph node metastasis negative and positive group (negative vs. positive). The likelihood ratio and Pearson’s statistics were used to evaluate differences in the contingency tables. Univariate logistic regression was performed to evaluate the independent correlation between potential risk factors and ALN metastasis in breast cancer patients with one or two positive SLNs. Multivariate proportional hazard analyses were performed to select major significant variables, and the selection of variables was based on the forward stepwise method using a *P*-value threshold. The multivariate logistic analysis was used to create logistic models. OR and corresponding 95% CI were estimated for each factor. A *p*-value of < 0.05 (two-sided) was considered to be statistically significant. All statistical analyses were conducted using SAS 9.4 (SAS Institute, Inc., Cary, NC, USA). Mathematica 10.1 (Wolfram Research Inc., Champaign, IL, USA) was utilized to calculate risk values.

## Results

### Demographic and clinical characteristics of the study subjects

A total of 389 breast cancer patients who had one or two positive SLNs and fulfilled the eligibility criteria were retrospectively analyzed. Of the 389 cases, 174 cases were pathologically diagnosed and confirmed to have breast cancer with ALN metastasis (44.7%), while 215 (55.3%) individuals had no signs of ALN metastasis. The detailed demographic and clinicopathologic characteristics, as well as laboratory tests findings, are summarized in Table 1. The average age of the patients was 51 years and ranged from 24 to 85 years. The two groups exhibited significant differences in age distribution, Her-2 status, and ratio of positive to total SLNs (*P* < 0.05), whereas the remaining characteristics including delay in diagnosis, tumor primary site, tumor location, tumor size, pathological type, ER status, PR status, triple negative status, Ki-67 status, tumor grade, and postoperative ALNs were not significantly different between the two groups (*P* > 0.05).

**Table 1:** Sociodemographic and clinical characteristics of the study subjects

N (%)		Patients with one or two SLNs (*n* = 389)			
Variable	Characteristics	Negative ALN (*n* = 215)	Positive ALN (*n* = 174)	x^2^/Z	*P*
**Demographic**					
**X1**	**Age**				
	< 60	162 (75.3)	146 (83.9)	4.2734	0.0387
	≥60	53 (24.7)	28 (16.1)		
X2	Occupation				
	Manual labor	96 (44.7)	95 (54.6)	4.9281	0.0851
	Mental work	51 (23.7)	40 (23.0)		
	Other	68 (31.6)	39 (22.4)		
X3	Menarche^a^				
	< 15	148 (68.8)	110 (63.2)	1.6259	0.2023
	≥15	52 (0.24)	52 (29.9)		
X4	Menopausal status				
	Premenopausal	121 (56.3)	98 (56.3)	0.0001	0.9933
	postmenopausal	94 (43.7)	76 (43.7)		
**Clinical Characteristics**					
X5	Delay in diagnosis (days)^a^				
	0–90	86 (40.0)	68 (39.1)	0.0168	0.9866
	91–180	49 (22.8)	39 (22.4)		
	181–365	28 (13.0)	29 (16.7)		
	> 366	51 (23.7)	37 (21.3)		
X6	Tumor primary site				
	Right	119 (55.3)	80 (46.0)	3.3805	0.066
	Left	96 (44.7)	94 (54.0)		
X7	Tumor location^a^				
	Upper Outer	125 (58.1)	113 (64.9)	5.2315	0.1556
	Lower Outer	34 (15.8)	30 (17.2)		
	Upper Inner	41 (19.1)	19 (10.9)		
	Lower Inner	12 (5.6)	12 (6.9)		
X8	Tumor Size^a^				
	≤2 cm	89 (41.4)	75 (43.1)	0.0299	0.8626
	> 2 cm	112 (52.1)	91 (52.3)		
X9	Pathological type^a^				
	IDC	199 (92.6)	159 (91.4)	0.1822	0.6695
	Other	16 (74.4)	15 (8.62)		
X10	ER^a^				
	(−)	42 (19.5)	36 (20.7)	0.0614	0.8043
	(+)	169 (78.6)	136 (78.2)		
X11	PR^a^				
	(−)	66 (30.7)	55 (31.6)	0.0213	0.8839
	(+)	145 (67.4)	117 (67.2)		
X12	HER-2a				
	(−)	137	106	0.0303	0.8618
	(+)	38	28		
X13	Triple negative^a^				
	Yes	15 (6.98)	14 (8.00)	0.1933	0.6601
	No	188 (87.4)	148 (85.1)		
X14	Ki-67^a^				
	(low)	164 (76.3)	133 (76.4)	0.0521	0.8195
	(high)	47 (21.9)	36 (20.7)		
X15	Tumor Grade^a^				
	1	28 (13.0)	18 (10.3)	0.5895	0.5555
	2	73 (34.0)	59 (33.9)		
	3	114 (53.0)	95 (54.6)		
X16	Postoperative ALNs^a^				
	0–10	55 (25.6)	53 (30.5)	−0.0294	0.9765
	11–19	136 (63.3)	94 (54.0)		
	≥20	19 (8.8)	25 (14.4)		
**X17**	**Ratio of positive to total SLNs**^**a**^				
	< 0.5	155 (72.1)	86 (49.4)	20.4071	< 0.0001
	≥0.5	60 (28.0)	87 (50.0)		

*Abbreviations*: *ER* estrogen receptor, *PR* progesterone receptor, *Her-2* human epidermal growth factor receptor-2, *SLN* sentinel lymph node, *ALN* axillary lymph node, *IDC* invasive ductal carcinoma, *SD* standard deviation. ^a^Missing data: Menarche (*n* = 27); Delay in diagnosis (days) (n = 2); Tumor primary quadrant (*n* = 3); estrogen receptor (ER) (*n* = 6); Progesterone receptor (PR) (*n* = 6), Her-2 (*n* = 12); Ki-67 (*n* = 8); Tumor size (*n* = 22); Triple negative (*n* = 24); Tumor grade (*n* = 2); Ratio of positive to total SLNs (*n* = 1); and postoperative ALNs (n = 3). All lymph nodes were examined with hematoxylin and eosin (H&E) staining. Her-2 positivity was defined as a score of 3+ or ≤ 2+ combined with an application on fluorescence in situ hybridization (FISH). The molecular subtypes were determined according to the criteria by the World Health Organization

### Univariate logistic regression analysis of the risk factors associated with ALN metastasis

Univariate logistic regression analysis of the demographic and clinicopathologic parameters was used to explore ALN metastasis-associated risk factors in breast cancer patients with one or two positive SLNs (Table 2). The patient’s age, occupation, and the ratio of positive to total SLNs were found to be strongly associated with ALN metastasis (*P* < 0.05), whereas ALN metastasis was not associated with the delay in diagnosis, tumor primary site, tumor location, tumor size, pathological type, ER status, PR status, Her-2 status, triple negative status, Ki-67 status, tumor grade, or postoperative ALNs (*P* > 0.05).

**Table 2:** Univariate logistic regression analysis of the relationship between sociodemographic, clinical, and pathological factors and ALN metastasis in breast cancer patients with one or two positive SLNs

	Parameter	β	SE	***P***	OR	95% CI
**X1**	**Age**	**−0.5341**	**0.26**	**0.04**	**0.586**	**0.352–0.976**
**X2**	**Occupation**	**−0.2695**	**0.1221**	**0.0273**	**0.764**	**0.601–0.970**
X3	Menarche	0.2967	0.233	0.2029	1.345	0.852–2.124
X4	Menopausal status	−0.00174	0.2056	0.9933	0.998	0.667–1.494
X5	Delay in diagnosis (days)	−0.00233	0.086	0.9784	0.998	0.843–1.181
X6	Tumor primary site	0.3760	0.2048	0.0664	1.457	0.975–2.176
X7	Tumor primary quadrant	−0.1329	0.1088	0.2218	0.876	0.707–1.084
X8	Tumor Size	−0.0365	0.2109	0.8626	0.964	0.638–1.458
X9	Pathology	0.1599	0.3748	0.6697	1.173	0.563–2.446
X10	ER	−0.0632	0.2547	0.804	0.939	0.570–1.546
X11	PR	−0.0323	0.2209	0.8839	0.968	0.628–1.493
X12	HER2	−0.0487	0.2806	0.8621	0.952	0.549–1.651
X13	Triple negative	−0.1542	0.3865	0.69	0.857	0.402–1.828
X14	Ki-67	−0.0570	0.2503	0.8199	0.945	0.578–1.543
X15	Tumor grade	0.0994	0.1481	0.502	1.105	0.826–1.476
X16	Postoperative ALNs	−0.0632	0.089	0.4778	0.939	0.789–1.118
**X17**	**Ratio of positive to total SLNs**	**0.9606**	**0.215**	**< 0.0001**	**2.613**	**1.715–3.983**

*Abbreviations*: *OR* odds ratio, confidential interval, *ER* estrogen receptor, *PR* progesterone receptor, *Her-2* human epidermal growth factor receptor-2, *SLN* sentinel lymph node, *ALN* axillary lymph node. To avoid missing any significant indicators, the factors with a significance of *P* < 0.05 in the univariate logistic regression analysis, including age, occupation, and the ratio of positive to the total number of SLNs were considered to entry into the multivariate logistic regression analysis

### Multivariate logistic regression analysis of risk factors associated with ALN metastasis in breast cancer patients with one or two positive SLNs

The demographic, social, and clinicopathological characteristics, as listed in Table 1, were also used for the multivariate logistic regression analysis to determine independent risk factors for prediction of ALN metastasis in the breast cancer patients with one or two positive SLNs. The resulting data were expressed as ORs with 95% CIs. As shown in Table 3, age (OR 0.612, 95% CI: 0.379–0.989), the ratio of positive to total SLNs (OR 2.603, 95% CI: 1.765–3.838), and occupation (OR 0.786, 95% CI: 0.626–0.985) were found to be significantly different between the two groups (positive versus negative ALN metastasis), and these risk factors were independently associated with ALN metastasis in breast cancer patients with one or two positive SLNs (*P* < 0.05).

**Table 3:** Multivariable logistic regression analyses of the risk factors associated with ALN metastasis in breast cancer patients with one or two positive SLNs

	Parameter	β	SE	***P***	OR	95% CI
	Intercept	−0.5262	0.4468	0.239		
X1	Age	−0.4902	0.2447	0.0451	0.612	0.379–0.989
X2	Occupation	−0.2414	0.1155	0.0365	0.786	0.626–0.985
X17	Ratio of positive to total SLNs	0.9565	0.1982	< 0.0001	2.603	1.765–3.838

*Abbreviations*: *OR* odds ratio, *CI* confidential interval, *SLN* sentinel lymph node, *ALN* axillary lymph node. Logit(P) =  − 0.5262 − 0.4902X_1_ − 0.2414X_2_ + 0.9565X_17_

## Discussion

Breast cancer patients who undergo the ALND procedure often experience serious complications associated with the surgery. To date, it is still debated if ALND should be avoided in breast cancer patients with one or two positive SLNs [13, 26–29]. Currently, the recommendation often relies on whether the patient presents with ALN metastasis. Until now, the incidence of ALN metastasis in breast cancer patients with one or two positive SLNs has not been largely investigated and the benefit of ALND has not been established in Chinese breast cancer patients with one or two positive SLNs.

In this study, we evaluated Chinese breast cancer patients with one or two positive SLNs who undergone ALND with or without ALN metastasis. There were four primary findings from this study. (1) Of the breast cancer patients with one or two positive SLNs, a smaller proportion of the study subjects had ALN metastasis (Table 1); (2) univariate logistic regression analysis showed that age, Her-2 status, and the ratio of positive to total SLNs were significantly associated with ALN metastasis (Table 2); (3) multivariate logistic regression analysis showed that age, occupation, and ratio of positive to total SLNs differed significantly between patients with and without ALN metastasis, suggesting that they were independently associated with ALN metastasis (Table 3); and (4) breast cancer patients with one or two positive SLNs who were 60 years of age or older, performed mental labor jobs, and had a lower ratio of positive to total SLNs showed low likelihood for developing ALN metastasis, allowing for the ALND procedure to be omitted from the treatment strategy. However, the risk for positive ALN was higher in breast cancer patients with one or two positive SLNs who were less than 60 years of age, performed manual labor jobs, and had a higher ratio of positive to total SLNs. In these patients, ALND was recommended for improved clinical outcomes.

The present study demonstrated that ALN metastasis was less prevalent in breast cancer patients with one or two SLNs in older women (≥60 years of age) than younger women (< 60 years of age). It has been well-documented that older age is a risk factor for many forms of cancer, including breast cancer [3, 30], with approximately 40% of breast cancer cases occurring in older women [30]. However, the breast tissues of older women show age-associated changes in the sensitivity to female hormones and often present with favorable histology and low-grade disease [31]. This alteration in estrogen sensitivity and other biological changes, such as tumor microenvironment modifications, immune senescence, and epithelial cells may be contributing factors [31]. In this study, we discovered that occupation types [manual labor jobs, mental labor jobs, and others (the rest)] affected the status of ALN metastasis as patients who performed manual labor jobs showed a higher incidence of ALN metastasis than those who performed mental work or other occupations (the rest). In addition, we identified the ratio of positive to all SLNs as an independent risk factor for ALN metastasis, as it was positively associated with a higher risk for ALN metastasis in breast cancer patient with one or two positive SLNs.

In this retrospective study, a proportion of the study patients showed a positive family history of breast cancer or other cancers in their first-degree relatives (FDRs: father, mother, sisters, or brothers). However, there was no significant difference between the ALN metastasis and non-metastasis groups.

In this study, we emphasized a Chinese patient cohort primarily because differences exist between Chinese and Western populations. Several previous studies suggested that the risk factors for breast cancer had differential effects on the ALN metastasis among the different populations. Çetintaş and colleagues have reported that other independent risk factors, including the presence of perineural invasion, lymphatic vessel invasion, age of < 40 years, and an extensive intraductal component (> 25%), were significantly associated with an increased risk of ALD metastasis in breast cancer patients [32]. In particular, Liu and colleagues reported distinct tumor characteristics between Chinese and Western breast cancer patients, including that the number of isolated tumor cells (ITCs) and micrometastases in Chinese breast cancer patients was much lower compared to Western breast cancer patients [33]. Until now, the risk factors were identified, and diagnostic nomograms were developed mainly in the Western population. Considering these differences, we strongly believe that there is a need for risk factors to be investigated in Chinese women by applying their own sociodemographic factors to better represent the risk factors associated with ALN status in Chinese breast cancer patients with one or two positive SLNs.

In this study, the occupation was categorized into manual and mental labor jobs. In contrast to mental labor jobs, responsibilities in manual or physical labor jobs involve more physical or laborious tasks using their muscle power. Previous studies on occupation and risk of breast cancer have indicated variations in the risk of developing breast cancer between two occupational groups: white-collar and blue-collar females [34–36]. White-collar women who worked as teachers, librarians, or administrative jobs were at a higher risk of developing breast cancer than blue-collar women [34–36]. The causes underlying this difference are unknown, yet it has been speculated that late first-time pregnancy might contribute to the difference. In the present study, to assess an association between occupation and the incidence of ALN metastases, occupation was categorized into manual and mental labor jobs, and the study patients were subdivided into three subgroups: manual labor jobs, mental labor jobs, and others/rest.

Under the presence of the three independent factors, including younger age (< 60 years), manual worker, a higher ratio of positive to total SLNs, patients experienced an increased risk for development of ALN metastasis. Oppositely, older age (≥60 years), mental labor jobs, and a lower ratio of positive to total SLNs were indicative of a lower likelihood of ALN metastasis, which meant that ALND could be unnecessary or could have been avoided in these breast cancer patients. Thus, the findings of this study may aid in the treatment decision-making process, leading to better clinical outcomes. In this study, we integrated a delay in the diagnosis of breast cancer into both univariate and multivariate logistic regression analyses, as the diagnosis delay with the time between the onset of symptoms and the initial medical consultation, professional delay between the initial consultation and the initial treatment, and between the time from the initial onset of symptoms to the initial treatment were previously found to be associated with the prognosis of cancer patients [37, 38]. In our study, various time periods in the diagnosis delay were divided into four categories, including 0–90, 91–180, 181–365, and ≥ 366 days, which were used in the univariate and multivariate logistic regression analyses. The resulting data did not exhibit any significant correlations between the delay in the diagnosis of breast cancer and the status of ALN metastasis.

The American College of Surgeons Oncology Group (ACOSOG) Z0011 trial has shown that ALND offers no additional benefit and may not be necessary in some breast cancer patients, suggesting the change of clinical ALN surgery management for eligible breast cancer patients. We noticed that the Z0011 clinical trial had specific criteria (e.g., T ≤ 5 cm, breast-conserving surgery, SLNs ≤2, postoperative radiotherapy) for omission of ALND, which would not be feasible for breast cancer patients who did not meet the criteria. Therefore, the clinical significance of the Z0011 study remains controversial. There is still debate about the most appropriate approach to implement the findings of the Z0011 trial into clinical practice, and the benefit of ALND in breast cancer patients with 1–2 positive SLNs remains inconclusive in the post-ACOSOG Z0011 trial era. Our study has identified independent risk factors for ALN metastasis in Chinese breast cancer patients with 1–2 positive SLNs, which allow for predicting the patients at low risk for ALN metastasis, who may not require ALND. Considering that in most centers in China, including ours, the current treatment remains ALND in breast cancer patients with positive SLNs, our results may assist clinicians in implementing or omitting ALND in clinical practice.

We realized that the present study has limitations. For example, this study was conducted using the data of breast cancer patients from a single hospital, yet prospective studies at multiple centers with different titers would better represent the entire population of breast cancer patients. We also realized that the small number of patients may have affected the statistical power of this study, and future studies should recruit larger patient populations. In addition, future studies should construct a predictive nomogram based on the multivariate logistic regression results, allowing them to externally validate the reliability and reproducibility of these findings. There are ongoing relevant studies in our center.

## Conclusions

In summary, the results of this retrospective study have suggested that age, the ratio of positive to total SLNs, and occupation are independent risk factors associated with the status of ALN metastasis in breast cancer patients with one or two SLNs. Our findings also suggest that, in older breast cancer patients (≥60 years) who performed mental labor jobs and had a lower ratio of positive to total SLNs (< 0.5), the likelihood of ALN metastasis was relatively low. In contrast, breast cancer patients who were younger (< 60 years of age), performed manual labor jobs, and had a higher ratio of positive to total SLNs could be at relatively high risk for ALN metastasis and the ALND procedure could be recommended in these patients to improve their overall survival.

## Data Availability

The datasets generated and analyzed during the current study are available from the corresponding author on reasonable request.

## Abbreviations

ACOSOG: American College of Surgeons Oncology Group
ALND: Axillary lymph node dissection
ALNs: Axillary lymph nodes
BMI: Body mass index
CI: Confidence interval
ER: Estrogen receptor
FISH: Fluorescence in situ hybridization
Her-2: Human epidermal growth factor receptor-2
H&E: Hematoxylin and eosin
IDC: Invasive ductal carcinoma
IHC: Immunohistochemistry
IRB: Institutional Review Board
ITCs: Isolated tumor cells
OR: Odds ratio
PR: Progesterone receptor
SD: Standard deviation
SLNs: Sentinel lymph nodes
SLNB: Sentinel lymph node biopsy
WHO: World Health Organization
